# Morphological, ultrastructural, and molecular description of *Unicapsula fatimae* n. sp. (Myxosporea: Trilosporidae) of whitespotted rabbitfish (*Siganus canaliculatus*) in Omani waters

**DOI:** 10.1007/s00436-015-4851-y

**Published:** 2015-12-23

**Authors:** Sarah H. Al-Jufaili, Mark A. Freeman, Volodymyr K. Machkevskyi, Abdulrahman Al-Nabhani, Harry W. Palm

**Affiliations:** Aquaculture and Sea-Ranching, Faculty of Agricultural and Environmental Sciences, University of Rostock, Justus-von-Liebig-Weg 2, 18059 Rostock, Germany; Laboratory of microbiology analysis, Fishery quality control center, Ministry of Agriculture and Fisheries Wealth, Al Bustan, Sultanate of Oman; Ross University School of Veterinary Medicine, Basseterre, St. Kitts, West Indies the Federation of Saint Kitts and Nevis; Department of Electron Microscopy, College of medicine, Sultan Qaboos University, Al Khoudh, Oman

**Keywords:** Myxozoa, *Unicapsula*, Esophagus, Siganidae, Arabian Sea, Oman, Molecular

## Abstract

Investigations regarding the parasite fauna of wild whitespotted rabbitfish *(Siganus canaliculatus)* Park, 1797 revealed white, spherical, loosely attached cysts measuring 896 (375–1406) μm in diameter in the inner endothelial wall of the esophagus and stomach. Mature spores inside these cysts corresponded to the original description of spores belonging to the genus *Unicapsula* Davis, 1924. *Unicapsula fatimae* n. sp. spores were 6.23 (5.60–6.60) μm in length and 6.80 (6.12–7.39) μm in width. The length of large polar capsule was 2.62 (2.18–2.97) μm and width was 2.65 (2.32–2.90) μm, and the extended large polar capsule filament length was 15.50 (11.71–19.99) μm. Transmission electron microscope images of the plasmodia revealed a complex cyst structure that was unique among other *Unicapsula* spp. Ultrastructural details of the host–parasite interface and developmental stages of a species from the *Unicapsula* genus are described for the first time. Histology of an infected esophagus revealed some abnormalities and changes in the host tissue around the infection site, including hypertrophy of host esophagus epithelial cells and hyperplasia of host glandular tubules. The parasite presented here has been added to the genus *Unicapsula* using comparative morphological analysis and ultrastructural investigations supported by 18S small subunit ribosomal DNA molecular analysis.

## Introduction

Myxosporea are a class of fascinating, microscopic, metazoan aquatic parasites belonging to phylum Cnidaria (Kent et al. 2001; Lom and Dykova 2006). Since their discovery, they have attracted much attention because of their mysterious and complex life cycles (Anderson et al. 2000; Marton and Eszterbauer 2011), enigmatic phylogeny (Whipps et al. 2004; Fiala 2006; Evans et al. 2010), and negative impact and pathogenicity on wild (Yokoyama and Itoh, 2005; Burger et al. 2008; Dykova et al. 2011; Buchmann et al. 2012) and cultured (Katharios et al. 2014; Tossavi et al. 2015; Yuan et al. 2015) fish stocks. Although extensively investigated among various marine and fresh water fish hosts, there are few valid records of myxosporean parasites infecting wild and reared siganids worldwide. These are limited to few records from the Egyptian, Saudi Arabian, and Israeli coasts of the Red Sea. The highly pathogenic *Zschokkella iceterica* was reported in the gall bladder of wild *Siganus luridus*, *Siganus rivulatus*, and *Siganus argenteus* in Israel (Diamant and Paperna 1986; Diamant 1992)*. Zschokkella helmii* was recorded from the gall bladder of *S. rivulatus* from the Red Sea, Egypt (Abdel-Ghaffar et al. 2008). Some unidentified ceratomyxids were observed from the gallbladder of *S. rivulatus* from Israel (Diamant 2010) and Egypt (Abdel-Ghaffar et al. 2008). An unidentified *Ortholinea* species from the urinary bladder of *S. rivulatus* caught off Israeli waters (Diamant 2010) and *Ortholinea saudii* was isolated from the kidney of *S. rivulatus* from the Kingdom of Saudi Arabia off the Red Sea (Abdel-Baki et al. 2015). To date, the only multivalvulid myxosporean reported from a siganid is *Kudoa iwatai*, a species known to cause systematic infection in cultured *S. rivulatus* from Israel (Diamant et al. 2005; Diamant 2010). Members of the genus *Unicapsula* (Davis 1924) are multivalvulids belonging to the family Trilosporidae, which accommodate myxosporean parasites that have three valves, each bearing a polar capsule (Lom and Dykova 2006). *Unicapsula* species are unique among other Trilosporidae because only one of the three polar capsules is fully developed and functional, whereas the remaining two are rudimentary and barely visible (Alama-Berjamo et al. 2009; Miller and Adlard 2013). Since the description of the genus and the type species in 1924 by Davis, a total of 12 species of *Unicapsula* have been recorded from different localities and a wide range of marine host species (Naidjenova and Zaika 1970; Schubert et al. 1975; Sarkar 1984; Sarkar 1999; Diebakate et al. 1999; Miller and Adlard 2013; Tomochi et al. 2014). Similar to their closely related group, the Kudoidae, some members of *Unicapsula* have been associated with negative impact on their hosts mostly associated with esthetic issues involving macroscopic pseudocysts or myoliquefaction (Lester 1982; Alama-Berjamo et al. 2009; Miller and Adlard 2013). Although the majority of species belonging to this genus has been detected from the musculature (Miller and Adlard 2013; Tomochi et al. 2014), some have been detected from other organs such as the gills (Diebakate et al. 1999), kidney (Sarkar 1999), and urinary bladder (Naidjenova and Zaika 1970). Although marine parasitological investigations in the Arabian Peninsula region dates back to the 1980s, the myxozoan parasite fauna received only most recent attention, resulting in several new species being recorded from various marine hosts, caught off the coasts of the Kingdom of Saudi Arabia (Red Sea and Arabian Gulf) (Zhang et al. 2014; Mansour et al. 2014, 2015a, 2015b). The present study describes a new species of *Unicapsula* using morphological, ultrastructural, histological, and molecular characterization, infecting the esophagus and stomach endothelium of *S. canaliculatus*.

## Material and methods

### Host sampling

Fish were bought as live or moribund from local fish markets and landing sites along the coast of the Sultanate of Oman from November to December 2012. Thirty five fish were obtained from Khasab landing site measuring 22.5–36.5 cm in total length and 140–562.2 g in weight, 35 fish from Daba local fish market (24.1–37.4 cm total length, 169.6–660.8 g in weight), 35 fish from Sohar local fish market (24.5–42.5 cm total length, 233.5–976.5 g in weight), 35 fish from Muttrah local fish market (31–39 cm total length, 320.3–690.5 g in weight), 35 fish from Masirah landing site (29.5–40.7 cm total length, 355–963.6 g in weight), 35 fish from Lakbi landing site (25.1–34.4 cm total length, 207.6–465.5 g in weight), and 35 fish from Raysut local fish market (29.1–41.4 cm total length, 320.6–801.9 g in weight). Once obtained, individual fish were immediately placed in plastic bags, labeled, and transported to the laboratory on ice (4 °C) or as immediately frozen samples (−20 °C). Fish were either examined directly after arrival in the laboratory or stored at −40 °C until further examination. Additional samples were obtained from fresh fish from Salalah (year 2013) and Muscat (year 2014) and examined immediately for histology and EM analyses.

### Parasitological examination and parasite collection

Thawed fish were dissected and all organs and body fluids were examined for presence of ecto- and endoparasites (Palm & Bray 2014). For detection of myxosporean parasites, microscopic slides were prepared from smears of the brain, liver, kidney, spleen, contents of gallbladder, and urinary bladder, and were initially observed at ×200–400 magnification using a Zeiss Axio Scope. A1 compound microscope. The esophagus, stomach, and intestine were cut open and examined for myxosporean cysts under Zeiss Stereo microscope (Discovery. V8). Gills were separated from the arches and observed under a stereomicroscope for cysts on the gill filaments. The operculum cover, buccal cavity, and abdominal cavity were examined through a magnifying daylight lamp at ×1.75 magnification (Daylight®). On the detection of cysts or free spores, their location and numbers (for the cysts) were noted and their dimensions were obtained.

### *Unicapsula* n. sp. spore morphology and measurements

**Fig. 1:**
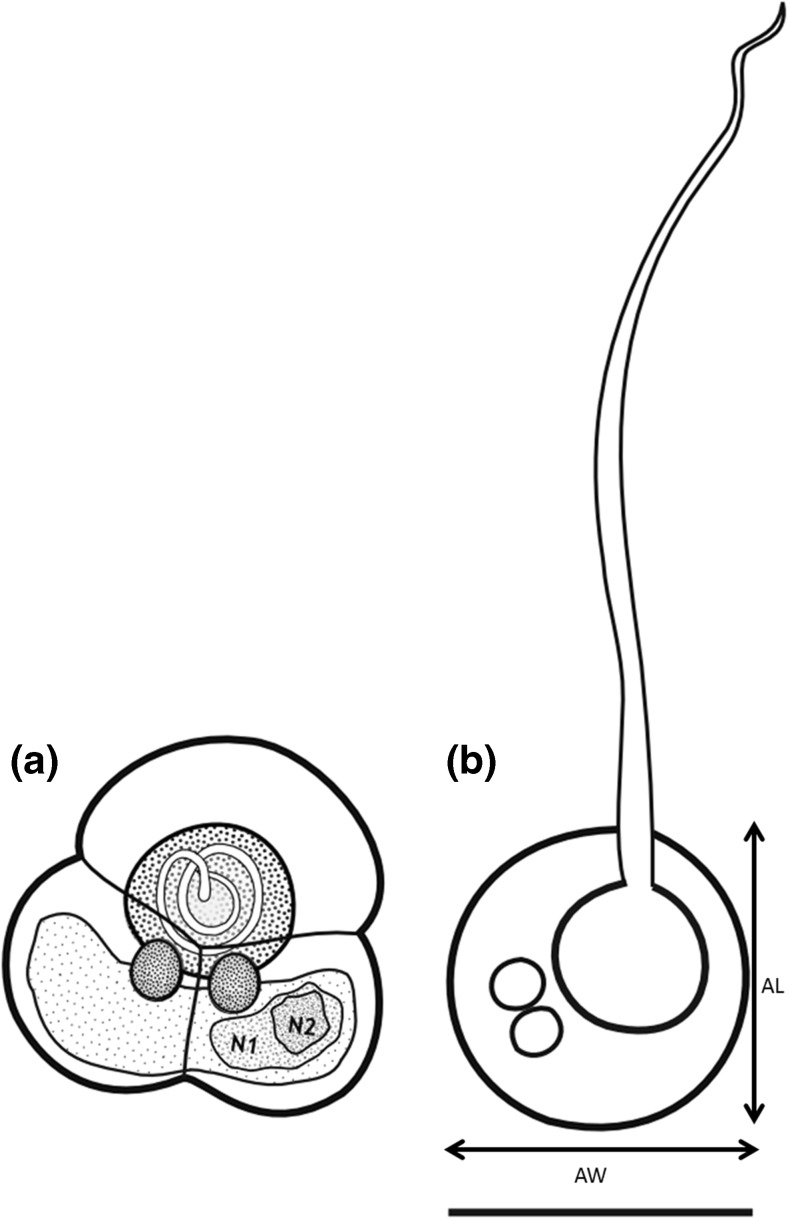
Line drawings depicting mature spores of *Unicapsula fatimae* n. sp. frontal view (**a**) and apical view (**b**). Scale bars **a** and **b** = 5 μm

Cysts that were detected from an infected esophagus were photographed, and their diameter was measured using a Zeiss stereo microscope (Discovery. V8) equipped with an AxioCam HRc digital camera, using AxioVision Rel. 4.8 software at ×1–×12 magnifications. Subsequently, individual cysts were separated from the infected tissues and a spore suspension was prepared by carefully disrupting the cysts using a sterile needle to release free spores in the physiological saline-filled small Petri dish (30 mm in diameter). A drop of prepared spore suspension was placed on a microscopic slide and was studied using an Olympus BX63 compound light microscope, equipped with an Olympus DP72 digital camera. Spores were observed using Nomarski differential interference contrasting illumination at magnification of ×200–1600, using oil immersion to study and describe the morphology of mature spores. Several photomicrographs were obtained using Olympus CellDimension© imaging software to obtain measurements of mature spores according to Alama-Berjamo et al. (2009). In addition, spore apical length and width were obtained as shown in Fig. (1b). Measurements of polar filaments were obtained using the polyline function to obtain the most accurate full length of the polar filaments. Because the rudimentary polar capsules of *Unicapsula* spp. are difficult to observe using light microscope, accurate measurements of the diameter rudimentary polar capsule were obtained from scanning electron microscopy (SEM) images only.

### Histology and host–parasite relationship

Infected and uninfected host esophageal tissues with parasite cysts were fixed in either 10 % buffered formalin or Bouin fixative for histological analysis of the host–parasite interactions. Tissues were processed using standard histological techniques and 5-μm thick sections were produced using a microtome. Sections were stained with hematoxylin and eosin (H&E) to study the host inflammation response, and cover slips were applied using DePex. Further slides were studied and photomicrographs were obtained and investigated for cyst structure, histopathology, and host–parasite interaction.

### Scanning electron microscopy imaging

For SEM, spore suspensions in physiological saline were centrifuged in 1.5 ml eppendorf tubes at 2000 rpm for 5 min to allow sedimentation of the spores to the bottom of the tube. After the supernatant was removed, pelleted spores were prepared for SEM as follows. The spore pellet was fixed in 2.5 % glutaraldehyde for a maximum of 3 h and was briefly vortexed to mix with the fixative. The fixed spore suspension was transferred to a syringe attached to a membrane filter holder and passed through a 13-mm diameter, 0.4-μm Whatman® nuclepore track-etch membrane filter, followed by two rinses in 100 mM sodium cacodylate buffer pH 7.2, each for 15 min. Further, the spores were post-fixed in 1 % osmium tetraoxide in 100 mM sodium cacodylate buffer for 30 min and were washed with distilled water for 15 min. The spores were dehydrated in an ascending series of ethanol (25, 50, 75, 95, and 100 %), each for 5 min. The membrane was removed from the holder, critical point dried, mounted onto aluminum stubs, sputter coated with gold, and viewed with a Jeol JSM 5600 LV SEM microscope at 60 Kv.

### Transmission electron microscopy imaging

Isolated cysts were fixed with 2.5 % glutaraldehyde in 1.0 M phosphate buffer (pH7.4) and were washed several times with the same buffer. The washed cysts were post-fixed in osmium tetraoxide in 1.0 M phosphate buffer and dehydrated in an ascending acetone series from 30 to 100 %. The cysts were embedded in epoxypropane by adding a 1:1 ratio of epoxy resin and acetone, 1:3 ratio of epoxy resin and acetone ratio, and full strength epoxy resin three times. The cysts were transferred to fresh resin in molds and dried for 48 h at 60 °C. Semi-thin sections were obtained from the cysts and were stained with 1 % toluidine blue for 1 min and mounted. Once the desired region of the cysts was observed, ultrathin sections were cut, mounted on grids, and stained with uranyl acetate and lead citrate. Grids were examined in a JEM 2100F field emission electron microscope (JEOL Ltd).

### DNA analysis and phylogeny

Infected tissues and parasite cysts were fixed in 95 % ethanol and given into DNA lysis buffer for molecular analyses. Total DNA was extracted using a GeneMATRIX DNA isolation kit (EURx, Poland) following the tissue protocol and used as templates in subsequent PCRs. Small subunit ribosomal DNA (SSU rDNA) of parasites was amplified using the general myxosporean primers according to the methodology described by Freeman et al. (2008) and the *Kudoa*-specific primers Kud-80f and Kud-730r (Kristmundsson and Freeman 2014), utilizing the same polymerase chain reaction (PCR) conditions. PCRs were conducted on parasite DNA from 4 fish and performed in triplicate. PCR products of the expected sizes were recovered using a GeneMATRIX PCR product extraction kit (EURx, Poland) and sequencing reactions were performed using BigDyeTM Terminator cycle sequencing chemistry, utilizing the same oligonucleotide primers that were used for the original PCRs. DNA sequencing was performed in forward and reverse directions for all PCR products, and nucleotide BLAST searches were performed for each sequence read to confirm a myxosporean origin (Zhang et al. 2000). Contiguous sequences were obtained manually using CLUSTAL X and BioEdit (Thompson et al. 1997; Hall 1999). CLUSTAL X was used for the initial SSU rDNA sequence alignments of the novel sequence and 19 other histozoic marine myxosporean parasites.

Phylogenetic analyses were performed using the maximum likelihood methodology in PhyML (Guindon et al. 2010) with the automatic smart model selection [selection criterion: Akaike Information Criterion (AIC)], running the general time-reversible substitution model (GTR + G6 + I) with 1000 bootstrap repeats.

## Results

**Fig. 2:**
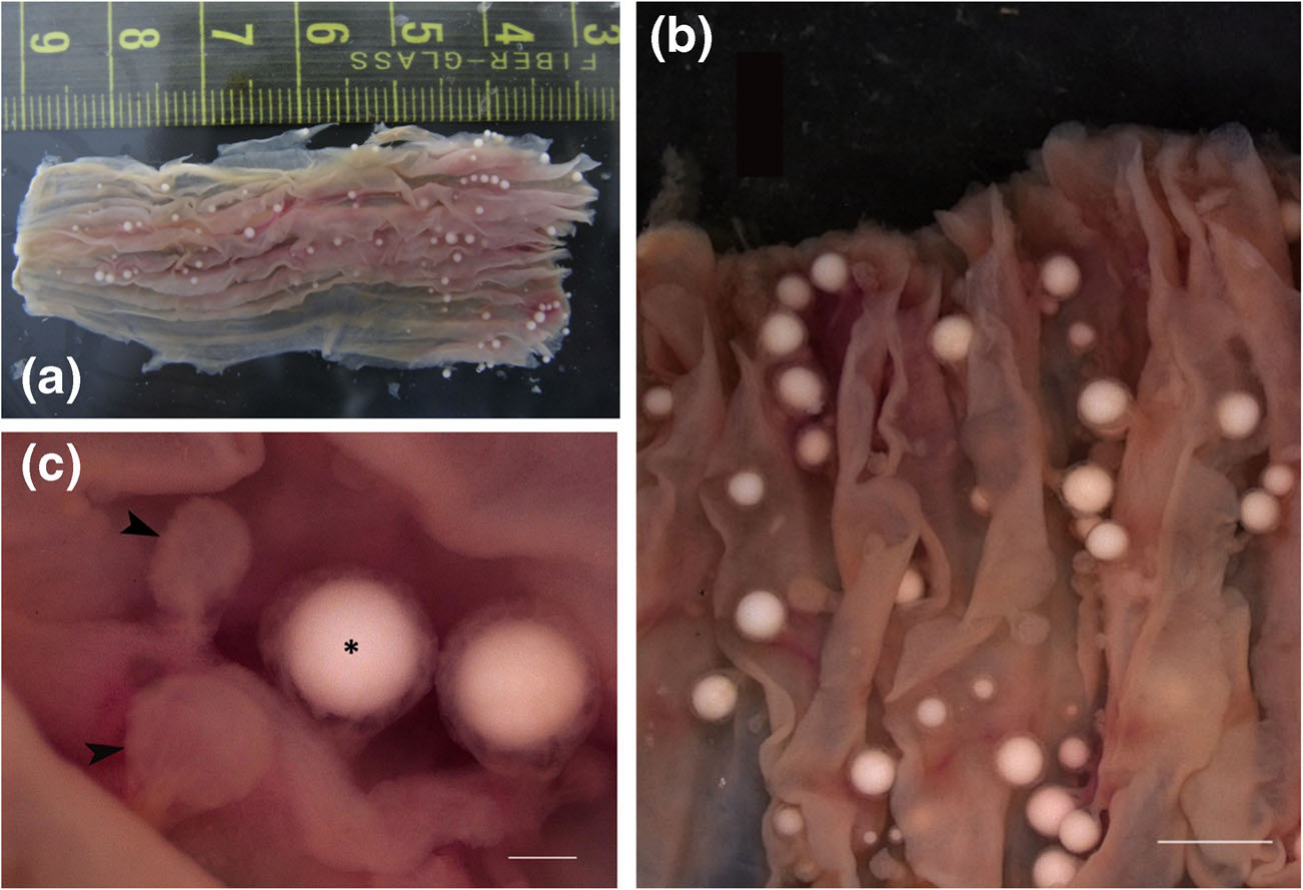
**a** Heavily infected esophagus of *Siganus canaliculatus* collected from Muttrah local fish market showing numerous *Unicapsula fatimae* cysts (>100 cysts detected). **b** Close-up of a portion of the infected esophagus showing the variable sizes of the cysts and several “empty” cysts. **c** Magnified portion of the esophagus showing two full cysts (*asterisk*) and two “empty” cysts (*arrow heads*). Scale bar 3 mm for **b** and 500 μm for **c**

Whitish, spherical, loosely attached cysts measuring 896 (375–1406) μm in diameter (*n* = 50) were detected from the esophageal and stomach inner lining of several *S. canaliculatus,* caught off Omani waters. The cysts contained myxosporean spores that had similarities to those from the genus *Unicapsula* Davis 1924 (Lom and Dykova 2006; Alama-Berjamo et al. 2009). The infection intensity ranged from 1 to 18 cysts per hosts (Fig. 2a–c, with numerous cysts are of a sample from Muscat Governorate collected in 2014). In some cases, several “empty” cysts were detected, which probably represented ruptured mature cysts (Fig. 2c). The cysts were detected in hosts from 5 out of 7 assigned sampling locations. The highest prevalence was from the Lakbi landing site with 17 of 35 examined fish infected.

### Taxonomical description

*U. fatimae* n. sp. from esophageal and stomach endothelium of whitespotted rabbitfish *S. canaliculatus* Park 1979.

Based on the information obtained from the shape of cysts, morphological, ultrastructural, molecular data of mature spores, site of infection, tissues tropism, host type, and geographical locality, we confirm that the *Unicapsula* species described herein is unique among the previously described *Unicapsula* spp.

**Class** Myxosporea

**Order** Multivalvulida

**Family** Trilosporidae Shulman 1959

**Genus***Unicapsula* David 1924

**Species***U. fatimae* n. sp.

**Type host:***S. canaliculatus* Park 1797 (Siganidae)

**Type locality:** Dhofar Governorate (Raysut City), Arabian Sea, The Sultanate of Oman

**Other localities**: Al-Wusta Governorate (Lakbi City), Al-Sharqiya Governorate (Masirah Island), Muscat Governorate (Muttrah City), and Musandam Governorate (Diba al Husn)

**Site of infection in the host**: Myxosporean cysts attached to the endothelium lining of host esophagus and stomach.

**Prevalence:** 11 out of 35 from Dhofar Governorate (31 %), 17 out of 35 from Al-Wusta region (48.6 %), 4 out of 35 from Al Sharqiya region (11 %), 3 out of 35 from Muscat Governorate (8.6 %), 0 out of 35 from Sohar city, 2 out of 32 from Diba (5.7 %), and 0 out of 35 from Khasab city off the Persian Gulf.

**Material deposited:** Glycerin–gelatin fixed spores on microscope slides MPM21011, MPM21012 and MPM 21013

**Etymology:** The species name, *fatimae*, is given in honor of my mother Fatima Al-Jufaili for her never-ending support and tireless care throughout my life.

**Fig. 3:**
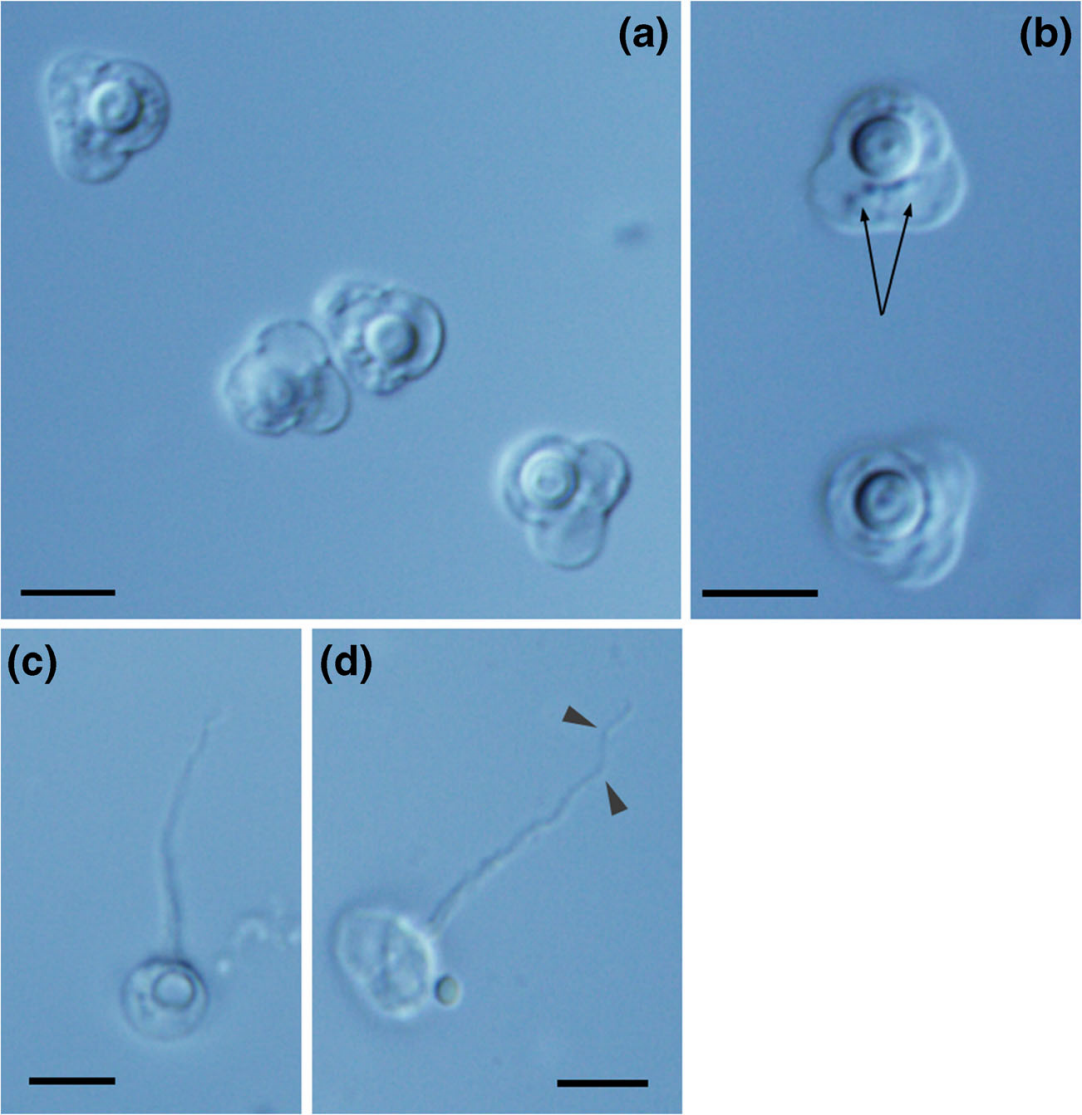
**a** Mature spores of *Unicapsula fatimae*. **b** Some mature spores of *U. fatimae* with visible rudimentary polar capsules (*arrows*). **c** Apical view of *U. fatimae* spores with extruded polar filament. **d** Extruded polar filament of *U. fatimae* tapering to the anterior portion and with double turns (*arrow heads*). Scale bar for all images =5 μm

**Description:** The description is based on 41 individual mature spores from thawed material. Spores trifolium with one large functional semi-spherical polar capsule and two smaller rudimentary polar capsules that are sometimes visible using light microscope (Fig. 3b). Sutural lines, which are not easy to observe with light microscope, divide the spore into three valves, one slightly larger than the other two. Mature spores 6.23 (5.60–6.60) μm in length, 6.80 (6.12–7.39) μm in width. The large polar capsule length was 2.62 (2.18–2.97) μm and width was 2.65 (2.32–2.90) μm. The length of the extended large polar capsule filament (*n* = 26; Fig. 3c) was 15.50 (11.71–19.99) μm. Polar filament tapering sometimes double turns at the anterior part (Fig. 3d). Turns of the large polar capsule filament were partially visible at ×1000 magnification with oil immersion; however, the number of turns was difficult to detect. Additional measurements were obtained from the spore apical view (Fig. 1b); the apical length was 5.11 (4.45–5.76) μm and apical width was 5.41 (4.45–6.26) μm.

### Remarks

Morphological data obtained from this study showed that *U. fatimae* was morphologically comparable to what is termed as the “sub-spherical” *Unicapsula* species that include *Unicapsula andersenae* Miller and Adlard 2013, *Unicapsula seriolae* Lester 1982, *Unicapsula pflughfelderi* Schubert et al. 1975, *Unicapsula galeata* Naidjenova and Zaika 1970, *Unicapsula shulmani* Aseeva and Krasin 2001, *Unicapsula pacifica* Aseeva and Krasin 2001*, Unicapsula setoensis* Tomochi et al. 2014, and *Unicapsula chirocentrusi* Sarkar 1984, and was morphologically distinct from all other *Unicapsula* species. *U. andersenae* from *(Argyrosomus japonicus)* Temminck and Schlegel 1843*, (Acanthopagrus australis)* Günther 1859; (*Eleutheronema tetradactylum)* Shaw, 1804; *(Lutjanus russellii)* Bleeker 1849 and *(Sillago ciliata)* Cuvier 1829 from Australia was genetically the most closely related species to *U. fatimae* in the BLAST search with approximately 97 % similarity. However, spore size, diameter of the large polar capsule, and length of the polar filament of *U. andersenae* were considerably smaller compared with *U. fatimae.* In addition, the general shape of *U. andersenae* was more spherical compared with the trilfolium shape of the new species. Although the general shape of *U. seriolae* is very similar to *U. fatimae*, comparative morphological data revealed that the size of the polar capsule and polar filament of this species were much larger than those of *U. fatimae*. In addition, a comparison of the SEM images of the two parasites revealed that the rudimentary polar capsule of the former is located differently than in *U. fatimae*. Furthermore, molecular data, infection site, and type host differentiate *U. seriolae* from the new species. Spores of *U. pflughfelderi* were three fourth the size of *U. fatimae* and the length of the extended polar filament was one half compared with *U. fatimae*. In addition, host type, infection site, and species locality further distinguishes *U. pflugfelderi* from the new species. Because the description of *U. galeata* is poor, it was rather difficult to distinguish it from the new parasite species. However, superficial morphological comparison between the two species and site of infection for the new taxon (muscle tissue vs esophagus tissue and its host *(Parupeneus ciliatus*) Lacépède 1802 vs *(S. canaliculatus)* could be used to distinguish between the two species. Both *U. shulmani* and *U. pacifica* were excluded because of their larger spore size (*U. shulmani* 7.3–8.6 μm and *U. pacifica* 7.8–10.3 μm), type host *(Albatrossia pectoralis) *Gilbert 1892, infection site (*U. shulmani*: urinary bladder and *U. pacifica*: muscles), and geographical locality. The recently described *U. setoensis* had a slightly smaller spore size and shorter polar filament compared with *U. fatimae*. However, spore shape with the permanently extended polar filament and shell valve arrangement of *U. setoensis* separates it from all previously recorded *Unicapsula* species and from the new taxon described herein. Finally, *U. chirocentrusi* can be differentiated from the new species by the general shape of the mature spore in addition to its site of infection and type of host. With regard to the remaining *Unicapsula* species, they can be easily distinguished from *U. fatimae* by their unique spore shapes, infection site, and geographical locality (*U. pyramidata)* Naidjenova and Zaika 1970 and *( U. marquesi)* Diebakate et al. 1999, *(U. muscularis)* Davis 1924, and *(U. maxima)* Sarkar 1999). Because most of the *Unicapsula* species are very simple in their spore morphology with a few that exhibit unique features, there is a requirement to redescribe some *Unicapsula* species and to include ultrastructural and molecular data to better understand and differentiate previously described species and facilitate identification of new ones.

### Scanning electron microscopy

**Fig. 4:**
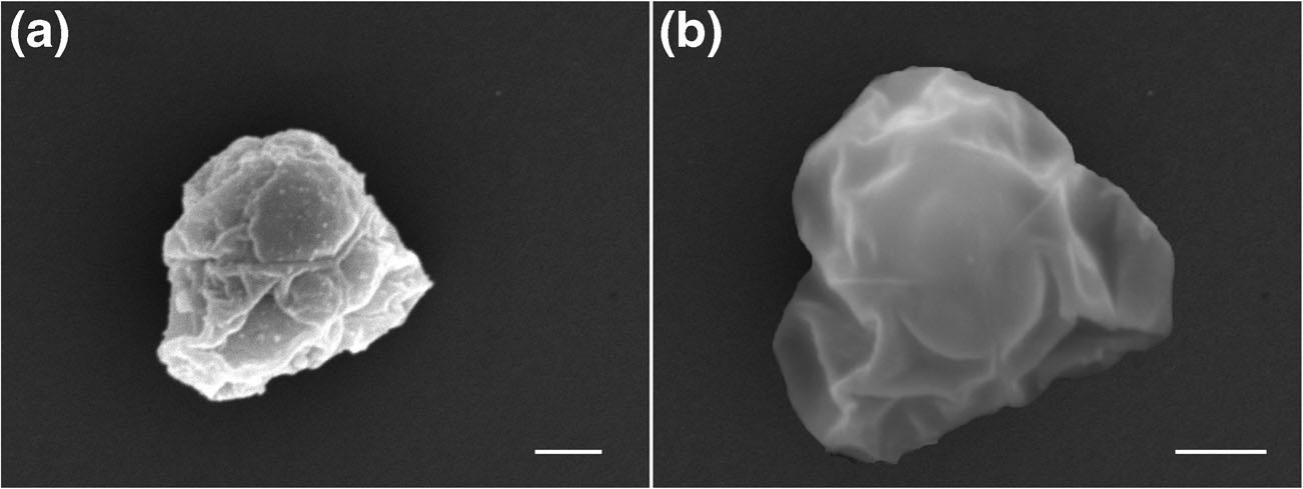
**a** SEM image of the apical pole view of a mature spore, showing the position of the large functional polar capsule and two rudimentary polar capsules immediately below it. The capsulogenic cells bearing the rudimentary polar capsule can be seen as two protrusions that take a leaf shaped form. The sutural lines form a Y shape on both the apical pole and basal pole view (**b**), dividing the three valves equally. Scale bar for **a**, **b** = 1.0 μm, 2.0 μm and 1.0 μm, respectively

The sutural line is clearly visible and forms a Y shape on the frontal and dorsal view, dividing the three valves almost equally (Fig. 4a). Rudimentary polar capsules were visible as leaf-shaped protrusion structures immediately under the large polar capsule, measuring 0.8 (0.7–0.9) μm in diameter. Compared with the only available SEM images of two *Unicapsula* species, the rudimentary polar capsules were similar to *U. pflugfelderi* Alama-Bermejo et al. 2009 in their shape and size.

### Transmission electron microscopy

**Fig. 5:**
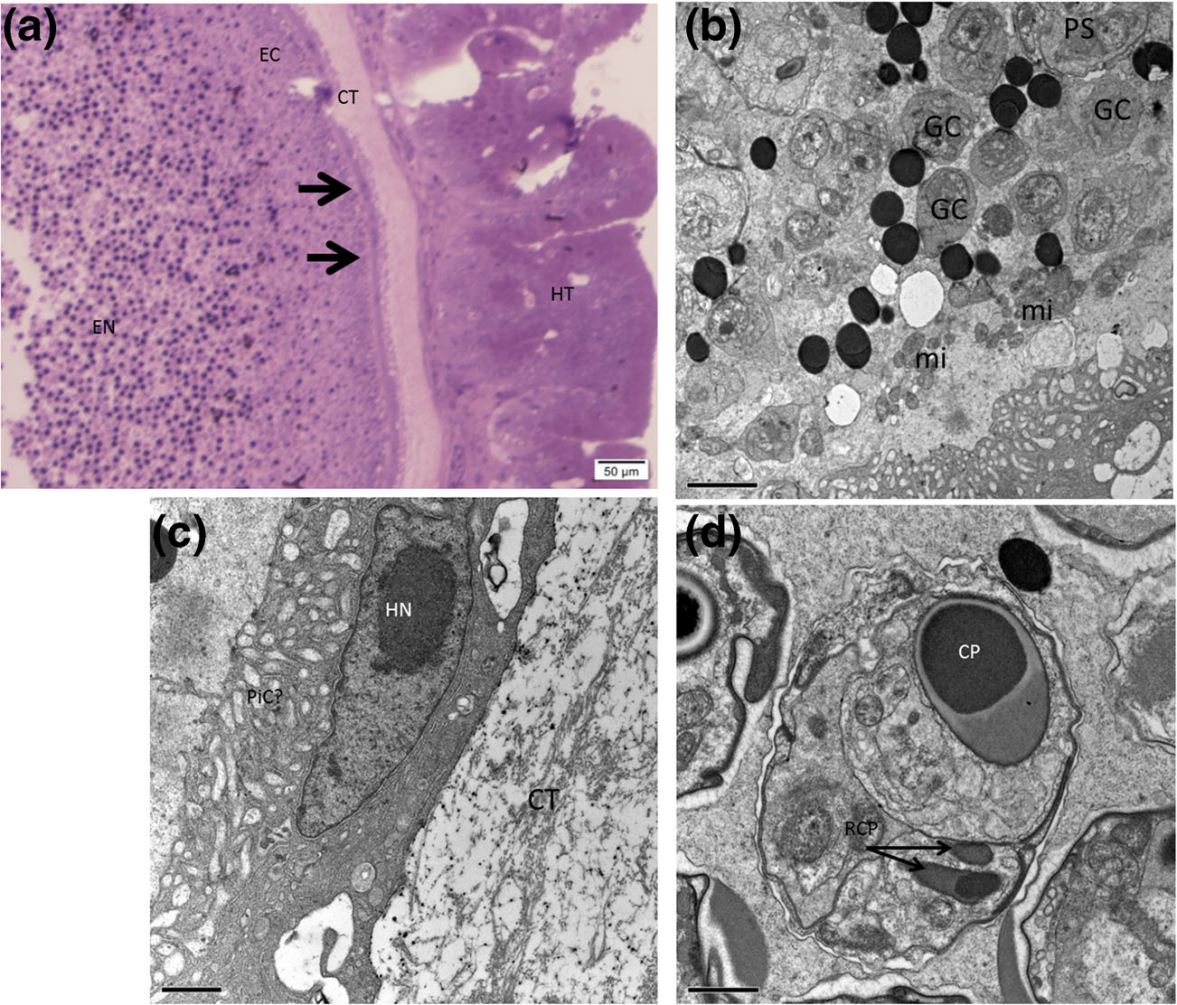
**a** Semi-thin section through a cyst showing the division of the cyst complex into several layers, the endoplasmic region (EN), ectoplasmic region (EC), peripheral membrane (arrows), connective tissue (CT), and host tissue (HT). **b** Ultrathin section of the plasmodia showing a close-up of the plasmodia and host interface with details of the peripheral membrane which is located between the ectoplasmic region and the connective tissue wall; note the host cell (*asterisk*) with the nucleus (HN) trapped inside the membrane and the several arms or web- like structure which possibly could be pinocytotic channels (PiC). **c** Details of the ectoplasmic region with several single nucleus *GC* generative cells, *PS* Pansporoblast, *mi* mitochondria, lipid droplets, and young spores. **d** Young spore with a developing polar capsule or *CP* capsular perimordium of the large polar capsule. Furthermore, note the two structures (arrows), which appear to be perimordium of the *RCP* rudimentary polar capsules. Scale bar for **b**, **c**, **d** = 1.0, 2.0, and 1.0 μm, respectively

**Fig. 6:**
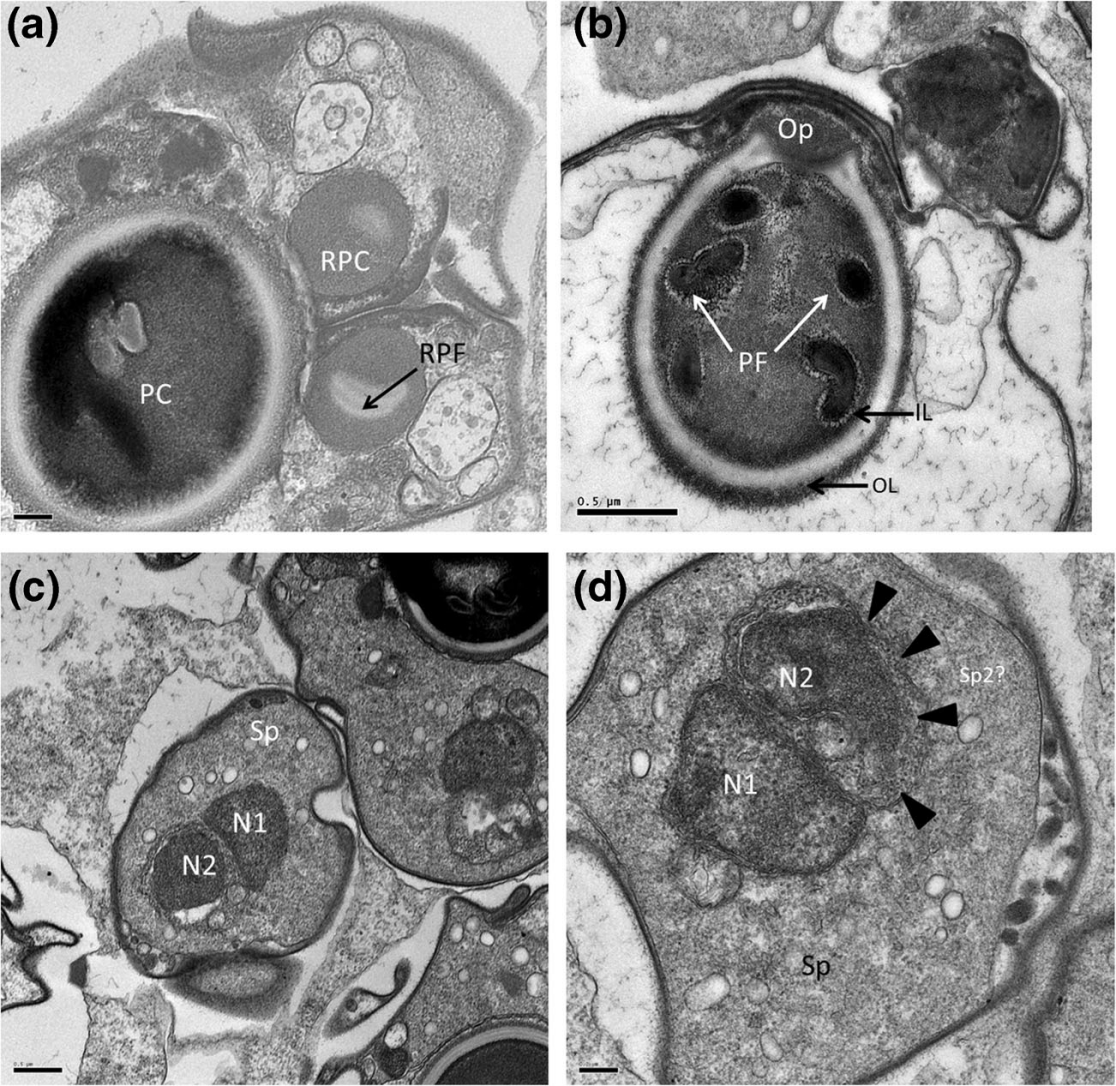
**a** Section through a mature spore showing the large polar capsule and the two rudimentary polar capsules with what appears to be a rudimentary polar filament. **b** Section through a mature polar capsule with the filaments showing 2 and half turns and the opening of the polar capsule. **c** Section through the sporoplasm of some mature spores showing the two adjacent nuclei. **d** Close-up of the sporoplasm showing the two nuclei and indicating the second membrane which could be a secondary sporoplasm (*arrow heads*) within the main sporoplasm. Scale bars: **a** and **d** = 0.2 and **c** = 0.5 μm

Semi-thin sections of the cysts revealed that they are divided into an endoplasm (EN; Fig. 5a) and ectoplasm (EC; Fig. 5a). A thin layer of darkly stained fibrous membrane was located outside the ectoplasm (arrows). A wall of host-originated connective tissue (CT; Fig. 5a) is observed surrounding the plasmodia and separating it from host epithelial tissue (HT; Fig. 5a); more details of the composition of host tissue complex surrounding the cysts is provided in the histology section). Ultrastructural details of the plasmodia observed using transmission electron microscope revealed more information regarding the composition of the membrane surrounding the ectoplasm (Fig. 5b). The membrane was a multilayered membrane unit that contained numerous branches or channels facing towards the ectoplasm. We assume that they could be pinocytotic channels (PiC) or passages that may aid in transporting nutrients into the plasmodia. These channels formed a web-like structure and contained several vacuoles of various sizes and shapes. In some areas, the host nucleus can be observed to be trapped in the complex membrane (Fig. 5b). Immediately next to the complex membrane, the ectoplasmic region is observed containing several mitochondria and electron dense lipid droplets (Fig. 5c). Sporogenesis was asynchronous with both generative cells and pre-sporogenic stages located at the periphery of the plasmodia and mature spores at the center. The earliest stages of the parasites are single nucleated cells of various sizes (possibly generative cells). Pansporoblasts were also observed and young spores with capsular primordium and primordium of rudimentary polar capsules (Fig. 5d) were seen in the ectoplasm region of the plasmodia. Mature spores were composed of three shell valves, one containing a large polar capsule possessing a fully functional polar filament and two contained two bodies, which were similar to the polar capsule; however, they were much smaller and had reduced polar filaments (Fig. 6a). The polar capsule was composed of an inner lucent layer and an outer electron dense layer similar to other *Unicapsula* spp. and other myxosporean parasites. Electron dense bodies were detected near the opening of the polar capsule and anterior to the rudimentary polar capsules. It was not easy to determine the exact number of turns of the polar filament for this species; however, after studying several images, the number of turns was estimated to be between two and one half and three turns. The sporoplasm of this parasite contained what appeared to be two adjacent nuclei in a single sporoplasm (Fig. 6c). A closer look at the sporoplasm and the two nuclei revealed the presence of another membrane surrounding one of the nuclei, which we think could be the second sporoplasm, as mentioned by earlier authors (Schubert et al. 1975; Lester, 1982; Alama-Bermejo et al. 2009) (Fig. 6d).

### Plasmodia gross morphology and histology

**Fig. 7:**
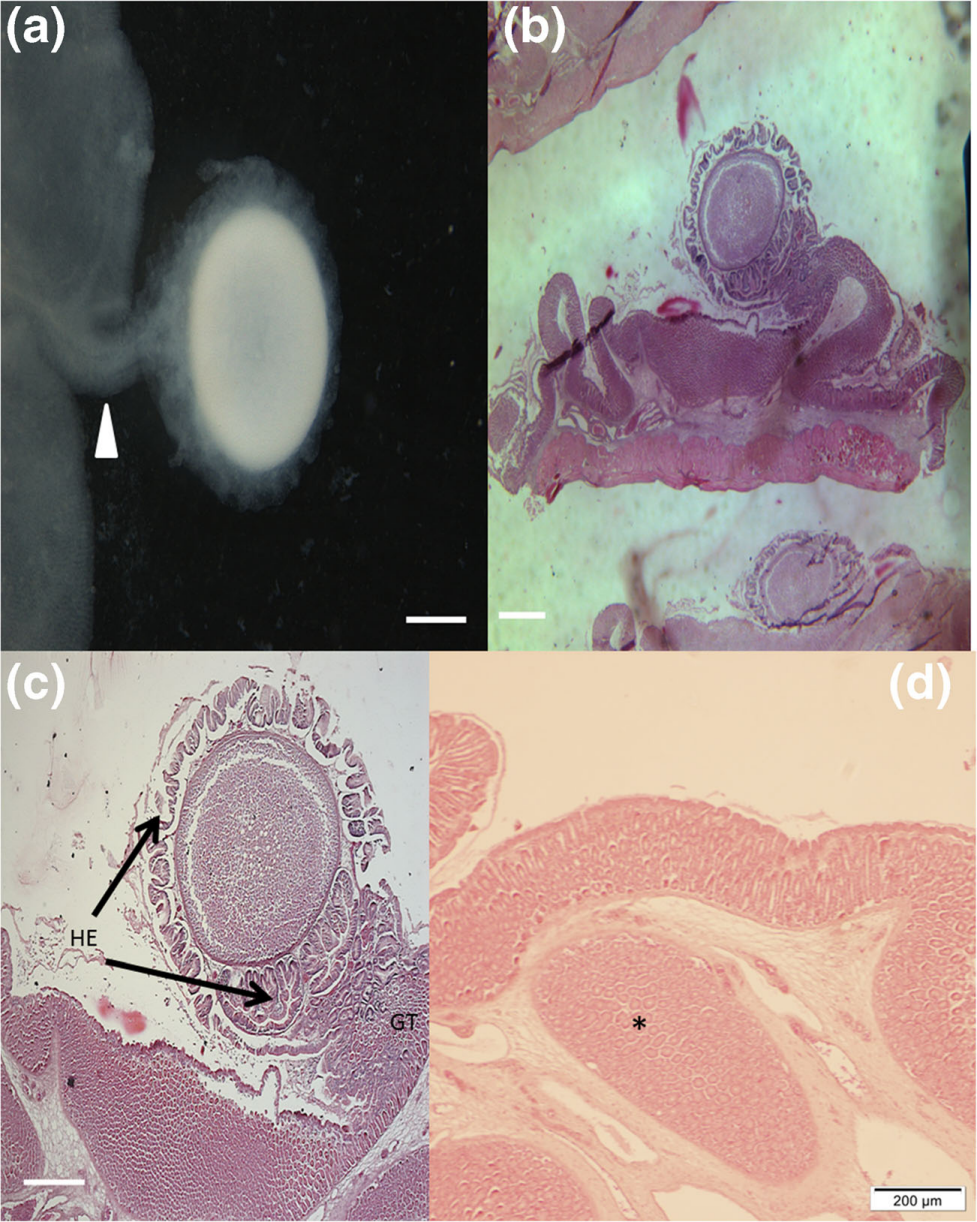
**a** Gross morphology of *Unicapsula fatimae* n. sp. cyst showing part of the host esophageal tissue, the stalk (peduncle) by which the parasite is attached on to the host tissue and the spherical plasmodia that is surrounded by host tissue (white arrow head). **b** Histological section through *U. fatimae* host complex showing the structure of the esophagus tissue near the infection site command position of the stalk structure and cyst complex. **c** Close-up of the cyst complex showing the hypertrophic folded host esophagus epithelial cells (HE) and glandular tubules (GT) that comprise the stalk formation. **d** shows the formation of an abnormal structure (asterisk) within the submucosal region of the esophagus. Scale bars: **a** = 500 μm, **b** = 600 μm, and **c** = 400 μm

The cysts of *U. fatimae* n. sp. were localized on the mucosal surface of the esophagus and occasionally on the gastric mucosa. The gross morphology shows that the cysts were attached to the host using a stalk that appears pedunculated (Fig. 7a, white arrow head). The cyst itself was surrounded with a layer of host-derived tissue, and appeared to be loosely attached to the inner lumen of the host esophagus (Fig. 7a). Histological sections of infected tissues with the cysts revealed that the cyst is composed of a multiple, complex structure consisting of a layer of folded hypertrophic host epithelial cells, a wall of host connective tissue, and a fibrous membrane separating the plasmodia from the host connective tissue. The function of the folded epithelial tissue layer is unknown, but its formation could be used by the parasite to protect itself and/or to maintain a supply of nutrients to the plasmodia. Histology of the “stalk” structure showed that it is composed of host epithelial cells, glandular tubules, and connective tissue. No inflammatory reaction could be detected in histological sections; however, some abnormalities were observed in the host tissue near the site of the infection. The mucosal tissue near the cysts was thicker than the normal tissue distant from the cysts and showed hyperplasia of glandular tubules and epithelial cells accompanied by hypertrophy of epithelial cells. In addition, it appears that the cysts begin developing within the submucosa and glandular tubule region of the host esophagus tissue. This observation is supported by observation of epithelial cells formed inside the glandular tubule regions and in some sections by the appearance of an “island” of host originated tissues (epithelial and glandular cells) observed in the submucosal region of the host esophagus.

### DNA and molecular analysis

**Fig. 8:**
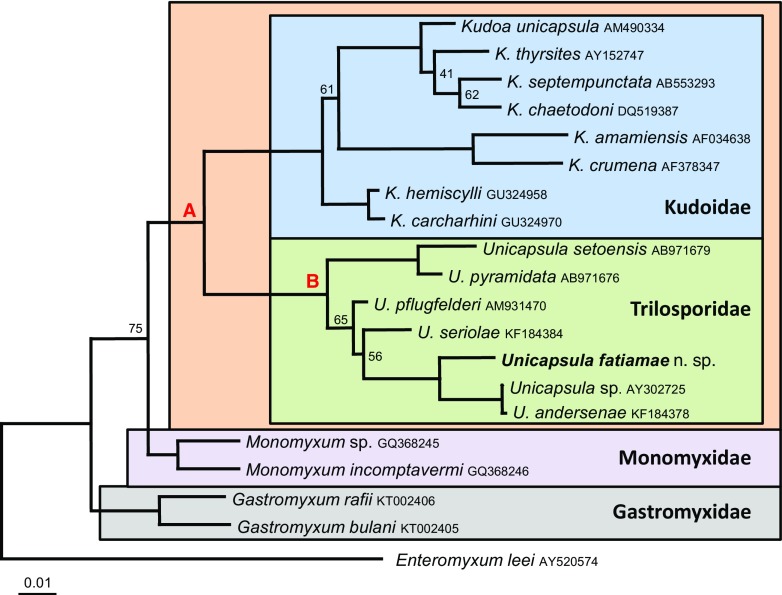
Maximum likelihood topology of 20 histozoic marine myxosporean SSU rDNA sequences using PhyML. Tree shows the phylogenetic relationship between the species of Unicapsula based on the available *Unicapsula* and the closest matches of 18s rDNA sequences available on GenBank NCBI. Numbers at the nodes represent bootstrap support values; nodes with no numbers are fully supported

An almost complete SSU rDNA sequence of 1653 bp was obtained for the *U. fatimae* sp. n. and the sequence was deposited in GenBank (Accession Number: KT894108). The sequences of the four strains of *Unicapsula* were 100 % identical. Submission to BLAST searches of the contiguous sequence showed that the closest relative in the databases were other *Unicapsula spp*. with similarities ranging between 90 and 98 %. The closest match was a sequence for *Unicapsula* sp. infecting the muscle of *Argyrosomus japonicus* from Australia (GenBank Accession No. AY302725). The SSU rDNA of 20 histozoic marine myxosporeans were used for phylogenetic analysis and the maximum likelihood topology was based on 1828 informative characters to produce a tree (Fig. 8). The resulting tree showed that the Trilosporidae are robustly supported from node A, as a monophyletic sister group to the Kudoidae and, together, they form the Multivalvulida. All available sequences of *Unicapsula* spp. are fully supported and form node B within Trilosporidae. The new taxon is placed in a fully supported sister clade with *Unicapsula fatimae* sp. and *U. andersenae* forming a monophyletic group. In addition, both *U. setoensis* and *U. pyramidata* formed another monophyletic sister group within the *Unicapsula*. Basal to the Multivalvulida are the Monomyxidae and Gastromyxidae, *Enteromyxum leei* was used as an outgroup.

## Discussion

A comprehensive morphometric comparison of all described *Unicapsula* species supports the view that the species presented in this article is a novel addition to the genus. This is the first record of the genus *Unicapsula* in Omani waters. Compared with previously described *Unicapsula* species, *U. fatimae* sp. n. is the only species among them to be found infecting the smooth muscles of a fish and the only one that forms spherical cysts. These two features alone strongly distinguish the new taxon from all previously described species. Further details of morphological features obtained through light microscopy and electron microscopy distinguish the new parasite from its congeners. Some ultrastructural details regarding *Unicapsula* species were provided by Schubert et al. (1975), Lester (1982), and Alama-Bermejo et al. (2009), both Schubert et al. (1975) and Alama-Bermejo et al. (2009) stated that the plasmodia of their respective *Unicapsula* species were divided into ectoplasmic and endoplasmic regions, whereas the ultrastructural description of Lester (1982) lacked such information. Similar to what has been described for the *Unicapsula* species and other histozoic myxosporeans (Ali et al. 2003; Azevedo et al. 2013), the plasmodia of *U. fatimae* sp. n. were divided into ectoplasmic and endoplasmic regions. However, unlike what is known from its congeners, it exhibited a more complex structure and contained cytoplasmic organelles and generative cells of the parasite, whereas Alama-Bermejo et al. (2009) described the ectoplasmic region of *U. pflughfelderi* as being smooth. In his description of *U. pflughfelderi*, Schubert et al. (1975) mentioned brief information with respect to the host–parasite interface, where he noted some channels near the periphery of the ectoplasm that were suggested to be pinocytotic channels. Investigations regarding the host–parasite interface in myxosporean infections in fish were reported extensively for the genera *Myxobolus* (Ali et al. 2003; Milanin et al. 2010) and *Henneguya* (Matos et al. 2005; Lovy et al. 2015). These studies report the presence of a fibrous single or double membrane unit with some pinocytotic activity at the periphery of the ectoplasm, and the appearance of several vesicles or vacuoles within the membrane and observation of some host cells in the vicinity of the membrane. For the first time, the present study presents some details regarding the host–parasite interface membrane reported from the genus *Unicapsula*. Similar to what has been reported by these authors, *U. fatimae* plasmodia had a fibrous membrane separating the plasmodia from the host tissue. The membrane of this parasite was complex and had a rather extensive network of web-like structures near the ectoplasmic region. Because many vesicles were observed on the membrane and supported by the occasional presence of host cells trapped in the membrane, *U. fatimae* plasmodia may feed off host cells via the process of pinocytosis.

Ultrastructure studies of *U. fatimae* plasmodia revealed information regarding the developmental stages of this new parasite that were similar to what is known to other myxosporean parasites (Ali et al. 2003; Adriano et al. 2009). This is the first description of developmental stages from a species in the genus *Unicapsula*. The earliest stages that could be seen were of single nucleated cells, which could be generative cells. From what was observed, it appears that sporogenesis was achieved by cell in cell development of generative cells to form a pansporoblast. This form of spore development was similar to what is observed in other myxosporean parasites (Abdel-Baki et al. 2015). The capsular primordium of the large polar capsule was observed in young spores often without any signs of formation of the rudimentary polar capsule. This could indicate that the formation of the larger polar capsules precedes the formation of a rudimentary polar capsule. Details of mature spores were in agreement to what has been described for other *Unicapsula* species (Schubert et al. 1975; Diebakate et al. 1999; Lester 1982 and Alama-Berjamo et al. 2009). The aforementioned authors recorded the presence of two sporoplasms in the respective *Unicapsula* species in their articles. In the present study, two nuclei were observed to be adjacent to each other, with one of them included in a membrane within the sporoplasm. We think that this is similar to what was described in the abovementioned studies, although slightly different. This feature of the sporoplasm may be a genus specific feature, which is exhibited only in *Unicapsula* species.

Histological investigations of the infected esophagus tissue did not reveal any inflammatory response induced by the parasite at the site of infection. However, the formation of the cyst complex and plasmodia peduncle induced some notable abnormalities in the host tissue; these changes could possibly impair the function of esophageal tissue. The possible negative impact of *U. fatimae* on its host could also be emphasized by the high intensity of parasites noted on some hosts with >100 cysts recorded in one host (samples collected from Muttrah city in 2014). In addition, the occurrence of several “empty” cysts indicates the release of mature spores into the lumen of the host esophagus, suggesting a possibility of parasite dispersion within the same host in the case of a direct life cycle. Further histological investigations are required to confirm the impact of this parasite on the host. Because *S. canaliculatus* is intended as a suitable candidate for the aquaculture industry in the Sultanate of Oman, a high prevalence and intensity of this parasite could be a possible to the future development of sustainable aquaculture in Oman.
